# Perceptions and Acceptability of Text Messaging for Diabetes Care in Primary Care in Argentina: Exploratory Study

**DOI:** 10.2196/10350

**Published:** 2019-03-18

**Authors:** Daniela Moyano, Daniela Morelli, Marilina Santero, Maria Belizan, Vilma Irazola, Andrea Beratarrechea

**Affiliations:** 1 Institute for Clinical Effectiveness and Health Policy (IECS) Buenos Aires Argentina

**Keywords:** mobile phones, short message service, diabetes mellitus, public health, qualitative research

## Abstract

**Background:**

Engagement in self-care behaviors that are essential to optimize diabetes care is challenging for many patients with diabetes. mHealth interventions have been shown to be effective in improving health care outcomes in diabetes. However, more research is needed on patient perceptions to support these interventions, especially in resource settings in low- and middle-income countries.

**Objective:**

The goal of the research was to explore perceptions and acceptability of a short message service (SMS) text messaging intervention for diabetes care in underserved people with diabetes in Argentina.

**Methods:**

A qualitative exploratory methodology was adopted as part of the evaluation of a program to strengthen diabetes services in primary care clinics located in low-resource settings. The diabetes program included a text messaging intervention for people with diabetes. A total of 24 semistructured telephone interviews were conducted with people with diabetes.

**Results:**

Twenty-four middle-aged persons with diabetes were interviewed. Acceptability was considered adequate in terms of its actual use, frequency, and the role of texts as a reminder. We found that text messages could be a mediating device in the patient’s learning processes. Also, being exposed to the texts seemed to help bring about changes in risk perception and care practices and to function as psychosocial support. Another relevant finding was the role of text messaging as a potential facilitator in diabetes care. In this sense, we observed a strong association between receiving text messages and having a better patient-physician relationship. Additionally, social barriers that affect diabetes care such as socioeconomic and psychosocial vulnerability were identified.

**Conclusions:**

Our findings show positive contributions of a text messaging intervention for the care of people with diabetes. We consider that an SMS strategy has potential to be replicated in other contexts. However, further studies are needed to explore its sustainability and long-term impact from the perspective of patients.

## Introduction

### Background

Diabetes mellitus is a leading cause of death worldwide with marked regional variation [1], resulting in a significant public health problem [2,3]. The global prevalence of diabetes in adults was 8.8% in 2015 and is predicted to rise to 10.4% by 2040; 81.1% of undiagnosed persons live in low- and middle-income countries (LMIC) [4].

In Argentina, the prevalence of diabetes increased from 8.4% to 9.8% between 2005 and 2013. In addition, an increase in diabetes mellitus–related deaths was observed in people aged older than 25 years [5,6].

Persons with diabetes are advised to have periodic visits with health providers and engage in self-care behaviors such as following a diet, taking medications, engaging in regular physical activity, and self-monitoring blood glucose [7,8]. These aspects of diabetes self-management are essential to optimize diabetes care, improve health outcomes, and prevent long-term complications [9,10]. However, many people find these behaviors difficult to achieve and maintain [11]. In fact, only 3.8% of Latin American patients with type 2 diabetes included in the International Diabetes Management Practice Study (IDMPS) achieved the recommended treatment goals of glycated hemoglobin <7%, low-density lipoprotein cholesterol <100 mg/dL, and blood pressure ≤130/80 mm Hg [12].

Patients need support from health care professionals to achieve these goals and, given the increasing prevalence of type 2 diabetes in LMIC, there is a need for innovative and effective ways to deliver self-management support interventions [13] between clinical encounters in resource-constrained health care systems. In this sense, interventions delivered via mobile phone short message service (SMS) text messaging have the potential to improve care with chronic diseases like type 2 diabetes [14-16] because unlike other technologies, mobile phones have a high penetration among low-income groups.

Although mobile health (mHealth) interventions have been shown to be effective in improving health care outcomes in diabetes [17-20], evidence about the likely uptake, best strategies for patient engagement, efficacy or effectiveness, and costs should guide the adoption of new technologies. Research on mHealth implementation is limited, and further research into these issues is needed. Also, there are significant information gaps regarding long-term effects, participant and provider acceptance, behavioral outcomes, costs, and the risks of such interventions with a focus in LMIC [21].

Latin America is in the process of expanding information and communication technologies and seeing an increase in the mobile network penetration [22]. The high prevalence of mobile phone availability, access, and use in low-resource settings offers a context in which it is possible to use these devices to improve health care delivery.

This study was conducted as part of a program to strengthen diabetes care in primary care clinics that include an mHealth intervention to support diabetes care for underserved populations.

### Diabetes Care Program

A diabetes care program was implemented in 20 primary care clinics (PCCs) within the national public system network located in low-income settings from 5 departments of the province of Corrientes, Argentina [23,24]. These clinics provide health care services and essential chronic care medication free of charge to persons with diabetes living in the catchment area. The program was developed by the Institute for Clinical Effectiveness and Health Policy (IECS), an academic organization, in collaboration with the Ministry of Public Health of the Province of Corrientes.

The intervention implemented by the program lasted 12 months and included (1) primary care team training for the implementation of clinical practice guidelines, (2) development of a Diabetes Registry to monitor and follow the patients up at the clinics, and (3) a text messaging intervention tailored to patient characteristics.

A total of 947 persons with diabetes were enrolled in the Diabetes Registry, of whom 62.3% (590/947) were women and 92.9% (880/947) had type 2 diabetes. The majority (830/947, 87.6%) had access to a mobile phone and agreed to receive SMS text messaging. Participants received an average of 53 texts during the study period. The study protocol is reported elsewhere [24], and the results of the program evaluation will be reported in future publications.

### The Text Messaging Intervention

- One-way weekly texts were sent to people with diabetes included in the Diabetes Registry until the participant came in for the 12-month follow-up visit.
- Texts were developed and validated using a methodology that evaluated understanding and appeal of each SMS text message using a 7-item questionnaire [24,25].
- Educational messages and reminders to address issues related with adherence to antidiabetic treatment, lifestyle modification, diabetes education, and facilitation of clinical encounters with the primary care team were included.
- Texts were tailored to baseline patient characteristics addressed by primary care physicians at the clinics (see Multimedia Appendix 1 for examples of text messages).
- A Web-based platform was developed to deliver texts.
- Texts were customized according to baseline characteristics and were sent in a fixed order.

To our knowledge, no study has been published in the region describing the experiences of people with diabetes with a text messaging intervention operating in routine clinical practice in PCCs located in low-resource settings. The aim of our study was to explore the perceptions and acceptability of an SMS text messaging intervention for diabetes care in underserved people with diabetes in Argentina.

## Methods

### Design and Study Participants

A qualitative and interpretative phenomenological study [26] of participant perceptions, experiences, and opinions of an SMS texting intervention was conducted in accordance with qualitative research guidelines during the implementation of the diabetes care program [27,28].

We used a combination of convenience and saturation sampling to enroll participants in the program. Study participants were selected from the Diabetes Registry if they met the inclusion criteria: adults aged 18 years and older with a diagnosis of type 2 diabetes who received care from selected clinics, had access to a mobile phone, and received texts during the implementation of the program. We included participants from a variety of departments to guarantee geographical coverage. The final sample size consisted of 24 informants between ages 39 and 66 years.

### Data Collection

An independent research team comprising researchers from the IECS led the qualitative study. No health care personnel were involved in the recruitment or interviewing process. Between March and October 2017, semistructured telephone interviews (with an average duration of 30 minutes) were conducted with study participants.

Semistructured interview guidelines, adapted during the data collection process, were developed based on the study objectives, including sensitive questions to identify emergent themes (see Multimedia Appendix 1 for the semistructured interview guideline, available only in Spanish).

Data collection stopped when data saturation was reached for the dimensions found, and it was judged that no new significant or relevant information emerged from the interviews.

### Data Analysis

Written transcripts of the interviews, which compounded the unit of analysis, were classified and then codified according to the study objectives and the dimensions addressed, constituting a single corpus of information.

The written transcripts were entered into ATLAS.ti version 7 (ATLAS.ti Scientific Software Development GmbH) software combined with the manual technique of information coding. Analytical dimensions were identified as constructs for the description of findings.

Finally, data were abstracted and interpreted through content analysis [29]. As part of the analysis, direct quotations representative of the participants’ opinions were selected and included in this manuscript to illustrate our findings. In order to protect the identity of the informants we only provide information on age and gender.

### Ethics

This study was reviewed and approved by the Institutional Review Board of the Hospital Italiano de Buenos Aires (CIE No. 2641, 22/10/2015). Participation in the study was voluntary. All participants signed an informed consent, and the confidentiality of the information was guaranteed.

## Results

### Characteristics of the Study Participants

Twenty-four adults aged 39 to 66 years were interviewed; 54% (13/24) were women. Selected participants had similar sociodemographic characteristics to those included in the diabetes program (Table 1).

The median diabetes duration was 7 years (interquartile range 4 to 10). As regards comorbidities, 15 participants (62%) had hypertension, 10 (42%) had dyslipidemia, 15 (62%) were obese, and 5 (21%) had at least one macrovascular or microvascular complication. At the time of the interview each participant had received a mean of 55 text messages during the program.

### Findings Dimensions

**Figure 1:**
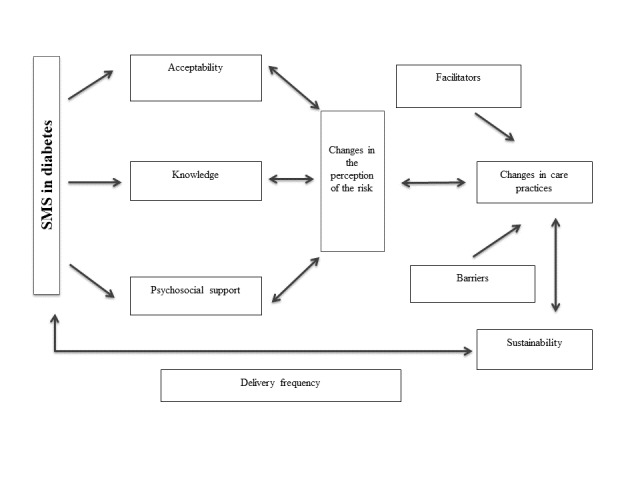
Emerging framework from our proposal.

From the analysis of participant discourse, we developed a qualitative framework about contributions of text messages to diabetes care (Figure 1). We included all the dimensions that emerged from the collected data regardless of the number of participants who mention them. We did not perceive differences in opinions associated to participant characteristics (clinic or sociodemographic).

**Table 1:** Sociodemographic characteristics of the population under analysis (N=24).

Characteristics		Value, n (%)
**Gender**		
	Female	13 (54)
	Male	11 (46)
**Age in years**		
	39-60	16 (67)
	>60	8 (33)
**Level of education**		
	7 years of schooling or less	13 (54)
	8-12 years of schooling	10 (42)
	>12 years of schooling	1 (4)
Health coverage, yes		12 (50)

### General Experience With the Text Messaging Intervention

The interviewed population gave some texts more attention than others (Multimedia Appendix 2). The most remembered messages were related to foot care: diabetic foot prevention, avoidance of infections, guidance on footwear, adequate foot care hygiene recommendations, and recommendations about when to see a physician.

Yes, [I remember] a message that was about the diabetic foot. It said that we have to wear footwear all the time because it is a silent illness.

Woman, 51 years

Messages about recommendations for a healthy diet were also well remembered. In this sense, study participants appreciated recommendations on the consumption of fruits and vegetables.

Messages to promote medical visits and foster compliance with diabetes medication and glycemic control were pointed out by participants less frequently. Messages about physical activity were not mentioned by participants.

### Acceptability of the Text Messaging Intervention

A set of characteristics was used to assess acceptability of the text messages. Components that emerged from the speeches analyzed were usability, frequency preferences, and a reminder function.

As for usability, participants indicated that they always opened and read the messages, and they ensured they had no problems with opening and reading them. Some participants expressed that they saved them in their phone devices or transcribed them in notebooks. They also stated that messages were very useful and that they felt happy and grateful to receive them. A weekly frequency of messages was perceived as adequate and well accepted. Additionally, a significant number of interviewees mentioned the reminder function of the text messages.

If there is one that interests me I’ll write it in a notebook...I keep the ones that I like the most in a notebook, so that I don’t forget.

Woman, 52 years

In addition, some factors were identified that might influence the sustainability of diabetes care such as the relationship established with the referring physician and proximity to the PCC (Multimedia Appendix 2).

### Text Messaging Impact on Knowledge About Diabetes

Subjective contents that emerged were related to changes in knowledge about diabetes before and after being exposed to text messages. The text works as a mediating device that facilitates the patient’s learning processes and promotes the dissemination of the acquired knowledge when messages are shared with family and friends (Multimedia Appendix 2).

### Text Message Contribution to Psychosocial Support

All participants expressed feelings associated with text messages during the course of their disease. Some reported that texts gave them a feeling of comfort and tranquility, and they valued the presence of text messaging as a confirmation that somebody remembers their illness and takes care of them without personally knowing them.

I feel more accompanied, I feel calmer. At least, someone who always remembers me because when you receive something in your cellphone, in your phone, you feel more comfortable, more peaceful.

Woman, 63 years

In addition, we observed that text messages impacted on the process of socialization of persons with diabetes. Furthermore, some interviewees reported that they shared text messages with a relative who also had diabetes, highlighting the role of texts as an educational device for transferring information (through oral communication and/or forwarding of messages; Multimedia Appendix 2).

### Effect of the Text Messaging Intervention on Changes in Risk Perception

Most of the interviewees mentioned some change in the perception of risk in diabetes after being exposed to text messages. This was reflected by emphasizing some associations between receiving messages and being more aware of diabetes care (Multimedia Appendix 2).

Yes, almost all [the messages] because it makes me aware, careful, and tells me how to take care of myself. We become conscious of what we suffer from.

Man, 59 years

### Effect of the Text Messaging Intervention on Changes in Diabetes Care Practices

Some changes in preventive and curative diabetes care practices were highlighted. We observed some changes regarding health care behaviors related to diabetes that could be linked to receiving text messages. These changes were mainly concentrated around healthy eating, weight loss, visits to the doctor, taking medication, physical activity, foot care, and attending medical supervision.

To a lesser extent, some patients stated that they were visiting their doctor more frequently. During these visits, they showed the doctor their sugar levels, had their feet checked, and had their vaccination scheme checked. Furthermore, the interviewees said that they now have better control of glycemia, glycated hemoglobin, and blood pressure values. Changes in physical activity, such as walking, were not much reflected in the perceptions of the interviewees.

Participants relayed that although they were willing to comply with diabetes care recommendations provided by text messaging, there were some barriers to diabetes care such as socioeconomic vulnerability, difficulties in accessing medical supplies (such as test strips or glucose meter) and healthy food, and psychosocial vulnerability (stress, conflicts in the family).

For me it’s fine, but the more support the better. You know why? Because I am a single parent, that is to say, mom and dad all in one...understand?

Woman, 47 years

Another relevant result is the role of the text message as a possible facilitator in the interaction with the PCC physicians regarding diabetes care. In this sense, some patients shared the messages they received with their physicians (Multimedia Appendix 2).

## Discussion

### Principal Findings

The qualitative approach used in this study allowed us to explore the perceptions of the people with diabetes that received a text messaging intervention. Our findings showed that a text messaging intervention with educational messages and reminders within the framework of a diabetes care program contributed positively to diabetes care and was accepted by people with diabetes with low educational level who live in low-resource settings.

### Comparison With Prior Studies

In agreement with our findings, Leon et al [30] showed that weekly texts were acceptable for persons with hypertension. Hacking et al [31] also found a positive attitude toward this intervention in patients with hypertension.

Messages that were recalled and remembered came from different domains. However, texts for foot care were the most remembered; this may be due to certain cultural valuations, previous knowledge, and the connotations that diabetic foot and its physical consequences [32-34], especially amputation, have in our society.

Exploring the most remembered messages for persons with diabetes allowed us to highlight the impact of messages with different content. This hierarchy, where informants gave some texts more attention than others, could be associated with certain cultural, symbolic, and evaluative patterns around the disease and its consequences.

In our study, participants perceived an increase in their knowledge of diabetes when exposed to text messaging. In accordance with previous studies [17,18], texts acted as a mediator in the patients’ learning processes facilitating the construction of significant learning [35].

The psychosocial support effect of text messaging in diabetes was important since it allows us to think about actions oriented toward a comprehensive approach to this chronic condition [36] and to contribute to the overall quality of life in persons with diabetes. Something similar was found in a study by Kwan et al [19]: texting services may help recognize distress and understand its effects on diabetes control, quality of life, and relationships with friends and family.

A distinctive fact that arises from this work and that has not been extensively addressed by other authors concerns the socialization effect of texts. In our study, informants stated that they shared the content of the texts with their family and friends. In this sense, we found that text messaging generates new ways of interaction, communication, and learning.

Risk perception is a critical determinant of health behavior [20]. In this regard, positive changes in risk perception were observed in patients after being exposed to text messages.

This was reflected when participants stated that they were more aware of diabetes when receiving the messages. Other studies that explored this effect of text messaging in conditions like HIV and in preventive programs [37,38] showed similar results.

Texts worked as a facilitator of the relationship between the people with diabetes and the health care team. Perhaps the texts alone are not responsible for this as text messaging intervention was implemented within the framework of a diabetes care program that also contemplated primary care training in diabetes management, education, and follow-up of patients. A relationship was observed between receiving texts and interacting with the referring physician. Similarly, this association was found in other studies aimed at patients with chronic diseases [30,36].

Social barriers to diabetes care were diverse. Although the participants interviewed received the texts adequately, we detected the presence of barriers that negatively impacted their self-management. These barriers (limited economic resources and lack of social support) have also been identified in other studies [39]. Additionally, qualitative study of cultural factors and diabetes found that social support may promote diabetes self-care but may also act as a barrier to diabetes management [32].

It is necessary to design interventions with an eye toward the limitations and context in which they will be implemented. Thus, qualitative research is a critical step in designing and implementing effective, feasible, and sustainable interventions.

Some studies have postulated that interventions with text messaging in LMIC have a positive impact on chronic disease management, including diabetes [14,40-43]. A qualitative perspective that takes the perception of patients is essential for the adoption and scaling up of these interventions. However, published studies on this subject are limited [32,44].

Several qualitative studies focused on other chronic noncommunicable diseases, such as hypertension, asthma, cervical cancer, and obesity, among others [30,44-47], in LMIC.

From the analysis of participant discourse, we developed a framework to explicitly theorize about contributions of texts to diabetes care. This theoretical framework has potential because it arises from the data. Also, our findings were similar to other frameworks previously published about chronic conditions based on the information, motivation, and behavioral skills model of health [48]. This leads to the need for greater knowledge production in this area, in particular from a diverse patient perspective [36].

### Strengths and Limitations

Among the limitations of this study may be those derived from qualitative research itself, such as lack of generalizability to other populations. We are aware that there may be a selection bias of respondents since the persons who responded may be more interested than others in the subject. However, we tried to minimize these biases by including persons of different ages, sex, and place of residence.

We were aware of the possibility of obtaining complacency bias as well. In order to minimize this bias, we used indirect and generic questioning, allowing respondents to project their own perspectives.

Finally, one of the strengths of our study was our exploration of subjective elements in persons with diabetes using a pragmatic and explanatory approach to better understand the experiential processes of this type of intervention.

### Conclusions

The study findings provide empirical evidence on the acceptability and value of text messages for diabetes care. We identified subjective elements of the SMS text message intervention such as adequate acceptability related to the frequency and content of the messages. Texts were found to be a source of diabetes knowledge and psychosocial support for people with diabetes. We also observed changes in risk perception and diabetes care practices. The knowledge gained in this study may reinforce the importance of adding an mHealth component like text messaging to programs for diabetes management implemented in low-income settings.

## Appendix

Multimedia Appendix 1 List of examples of short text messages and semistructured interview guideline.

Multimedia Appendix 2 Perceptions and acceptability about short message service.

## Abbreviations

IDMPS: International Diabetes Management Practice Study
IECS: Institute for Clinical Effectiveness and Health Policy
LMIC: low- and middle-income countries
mHealth: mobile health
PCC: primary care clinic
SMS: short message service
