# Supplementary material for: Perceptions and Acceptability of Text Messaging for Diabetes Care in Primary Care in Argentina: Exploratory Study
Source: JMIR Diabetes. 2019 Mar 18;4(1):e10350. doi: 10.2196/10350 (PMC6441856; doi:10.2196/10350)
Supplement: Multimedia Appendix 1 [file diabetes_v4i1e10350_app1.pdf]

**List of examples of short text messages**

| Domains | Spanish version of the SMS | English translation |
|---|---|---|
| Diabetes Information | La DIABETES es una enfermedad que no se cura y dura toda la vida. ¡Pero sí se puede controlar! Si está controlado, va a estar bien y llevar una vida normal. | DIABETES is a disease that does not heal and lasts for a lifetime. But it can be controlled! If it is controlled, you will be fine and lead a normal life |
| Diabetes Symptoms | ¿Sabía que cuando el azúcar en la sangre está alto puede marearse, ver borroso, sentir cansancio y orinar más seguido? Si le pasó esto, consulte a un médico. | Did you know that when your blood sugar is high you can get dizzy, see blurry, feel tired and urinate more often? If this has happened to you, consult with a doctor. |
| Goals | Para que su DIABETES esté bien controlada debe tener un valor de hemoglobina glicosilada de alrededor de 7 ¿Cuál es el suyo? | For your DIABETES to be well controlled, it must have a glycosylated hemoglobin value of around 7. What is yours? |
| Blood glucose monitoring | La única forma que tenemos de saber si nuestra azúcar en sangre está bien, es midiéndola ¿Usted se midió el azúcar? | The only way to know if our blood sugar level is good is by measuring it. Have you measured your sugar levels? |
| Diabetes medication-Adherence | Los medicamentos para la DIABETES funcionan muy bien y bajan el azúcar en sangre cuando los toma como le indicó el médico. | DIABETES medication works very well and lowers blood sugar levels when you take them as directed by the doctor. |
| Lifestyle-Physical Activity | En vez de ver tele, busque amigos o familiares que lo acompañen a caminar o a hacer otra actividad física. Eso lo hará fácil y entretenido. | Instead of watching TV, look for friends or family members to join you for a walk or any other physical activity. That will make it easy and entertaining. |
| Reminder-follow-up | Recuerde visitar a su médico al menos cada 3 meses para chequear cómo está su DIABETES. | Remember to visit your doctor at least every 3 months to check your DIABETES. |

**Semi-structured interview guideline**

**Cuestionario de aceptabilidad de SMS para participantes del estudio cualitativo**

**Código del participante: ________________**

**Fecha de la entrevista:**

**Encuestador: ________________**

"Buenos días/ buenas tardes, mi nombre es………………...soy investigadora y trabajo junto con el Centro de Salud (NOMBRE DEL CENTRO) en donde usted se atiende. En el centro donde usted se atiende se está desarrollando un programa para personas con diabetes. Uno de los componentes de este programa es el envío de mensajes de texto con información y consejos para que las personas con diabetes puedan cuidarse y estar mejor.

Lo estamos llamando porque nos gustaría conocer su opinión y hacerle algunas preguntas sobre los mensajes de texto que usted ha recibido por este programa.

1. **¿Alguna vez recibió mensajes de texto en su celular con información o consejos para cuidar su diabetes?**

SI ☐ 1

NO ☐ 2 **FINALIZA LA ENTREVISTA**

**Solo si responde SI a la pregunta 2.**

2. **¿Con que frecuencia, leyó los mensajes de texto en su celular con información o consejos para cuidar su diabetes?**

Nunca ☐ 1

Muy pocas veces ☐ 2

Algunas Veces ☐ 3

Casi siempre ☐ 4

Siempre ☐ 5

**3. ¿Siente que los mensajes de texto lo ayudan a cuidar su diabetes?**

SI ☐ 1 **2.1 ¿Cómo cree que lo ayudan?**

____________________________________________________________

____________________________________________________________

____________________________________________________________

NO ☐ 2 **2.2 ¿Por qué cree que no lo ayudan?**

____________________________________________________________

____________________________________________________________

____________________________________________________________

4. **Desde que recibe estos mensajes de texto ¿se sintió más acompañado?**

SI ☐ 1 **4.1 ¿Cómo cree que lo acompaño?**

____________________________________________________________

____________________________________________________________

____________________________________________________________

NO ☐ 2 **2.2 ¿Por qué cree que no lo acompañaron?**

____________________________________________________________

____________________________________________________________

____________________________________________________________

**5. ¿Recibir los mensajes lo hicieron estar más consciente de esta enfermedad?**

SI ☐ 1 5.1 **¿Por qué se sintió más consciente?**

____________________________________________________________

____________________________________________________________

____________________________________________________________

NO ☐ 2 5.2 **¿Por qué?**

____________________________________________________________

________________________________________________________________________

________________________________________________________________________

**6. ¿Qué mensajes le resultaron más útiles para cuidarse?**

**(De la siguiente lista puede decir todas las opciones que considere que fueron más útiles)**

Información sobre qué es la diabetes y cómo controlarla ☐1

Mensajes para acordarse de visitar al médico ☐2

Mensajes para acordarse de tomar su medicación ☐3

Información sobre la alimentación saludable ☐4

Información sobre actividad física ☐5

Consejos para cuidar el pie ☐6

Ninguno de los anteriores ☐7

Otro tema ☐8

Especificar: ________________________________________________

**6.1. ¿Recuerda algún mensaje en particular?**

7. ________________________________________________________________________

________________________________________________________________________

________________________________________________________________________

**8. ¿Está de acuerdo con la frecuencia de los mensajes de texto (una vez por semana)?**

SI ☐ 1

NO ☐ 2 ¿Por qué?________________

**9. Una vez que termine el Programa de Diabetes, ¿le gustaría continuar recibiendo mensajes de texto en su celular?**

SI ☐ 1

NO ☐ 2

**8.1 ¿Por qué?**

______________________________________________________________________
______________________________________________________________________
______________________________________________________________________

**10. ¿Le recomendaría a otra persona con diabetes incorporarse al programa para recibir los mensajes de texto?**

SI ☐ 1
NO ☐ 2

**9.1 ¿Porque?**

______________________________________________________________________
______________________________________________________________________
______________________________________________________________________

**11. ¿Tiene alguna(s) sugerencia(s) para este programa?**

______________________________________________________________________
______________________________________________________________________
______________________________________________________________________

**Ahora quiero hacerle una pregunta sobre el uso que usted le da a su celular…**

**12. De las siguientes opciones, ¿Qué uso suele darle a su celular?**
**(Puede decirme más de 1 opción)**

Hacer o recibir llamadas ☐1

Enviar mensajes de texto ☐2

Recibir mensajes de texto ☐3

Buscar información por internet ☐4

Usar Redes sociales como Facebook ☐5

Usar WhatsApp ☐6

**Observaciones:**

______________________________________________________________________________

______________________________________________________________________________

______________________________________________________________________________
