# Supplementary material for: Perceptions and Acceptability of Text Messaging for Diabetes Care in Primary Care in Argentina: Exploratory Study
Source: JMIR Diabetes. 2019 Mar 18;4(1):e10350. doi: 10.2196/10350 (PMC6441856; doi:10.2196/10350)
Supplement: Multimedia Appendix 2 [file diabetes_v4i1e10350_app2.pdf]

Dimensions	Categories	Characteristics / Descriptions	Findings	Verbatim
**1. General experience with SMS intervention**	*Recalled and remembered messages*	Where interviewees gave some SMS more attention to others is associated with cultural, behavioral, usage and thinking patterns around the disease and its consequences	Most remembered messages were related to foot care<br><br>Other messages well recalled:<br>• Healthy diet tips<br><br>SMS less recalled:<br>• medical visits reminders<br>• medication compliance reminders<br>• glycerin control<br><br>Messages about physical activity were not mentioned	"Yes, a message that was about the diabetic foot. It said that we have to wear footwear all the time because it is a silent illness." (Woman, 51 years)
**2. Acceptability of SMS intervention**	*Usability*	Interaction of user, system, and task in a specified setting and context	Reading and recording messages	"If there is one that interests me I'll write it in a notebook…I keep the ones that I like the most in a notebook, so that I don't forget" (Woman, 52 years)
	Frequency	Refers to the preferences regarding how often receive the SMS	Weekly reception, being adequate	"Yes, yes I'll gladly keep receiving the messages" (Man, 63 years)

	Reminder	Reminding care	The “reminder” function	“I’m happy because I forget, then I look at my cellphone, and the messages are there…I have them there…” (Man, 58 years)
	Sustainability	It refers to the ability to maintain the care compliance	Depends on the relation established with the referring physician and the proximity to the health care center	“Perhaps to help another person…to go with the doctor…” (Woman, 47 years)
**3. SMS impact on knowledge about diabetes**	*Knowledge in diabetes before and after SMS intervention*	It refers to the participants perception about the contribution of the SMSs on their learning about diabetes	A "mediating device" facilitates the learning processes	“I read all the messages to learn; there are things that I might not know and the messages could help me understand more about the illness” (Man, 61 years)
**4. SMS contribution to psychosocial support**	*Feelings of support*	Is related to the feeling of support and caring	The sense of comfort, tranquility, and the presence of someone who remembers their illness and cares for them without personally knowing them	“I feel more accompanied, I feel calmer. At least, someone who always remembers me because when you receive something in your cellphone, in your phone, you feel more comfortable, more peaceful…” (Woman, 63 years)
	Knowledge dissemination of SMS content	It refers to how the content of the messages can reach other community members	The patients that receive SMS considered themselves as outreach workers of knowledge.	“I always read it because my sister also has diabetes. When we get together for tea, I receive the messages, and I show her. I tell

			They read and share educational messages with family and friends	her that they're very useful for me…"(Woman, 53 years)
**5. Effects of the SMS intervention on risk perception**	*Perception about risk*	It refers to the subjective judgments about the likelihood of negative occurrences related with diabetes	Associations between receiving messages with increasing awareness regarding diabetes	"Yes, almost all (the messages) because it makes me aware, careful, and tells me how to take care of myself. We become conscious of what we suffer from…" (Man, 59 years)
**6. Effect of the SMS intervention on changes in practices in care**	*Self- care practices in diabetes care*	It refers to behaviors undertaken by people with diabetes to successfully manage the disease	These changes were mainly concentrated in six self-care practices: healthy eating; medical control; medicines; physical activity; foot care; assistance to the medical consultation	"The truth is that now I am more conscious, now I go more often to the "salita (clinic)"; before, I had to be called, now I go by myself. Besides, the girl in the "salita" tells me that I have to walk, watch what I eat, sometimes she tells me that she wants to check my feet, and she takes look…" (Woman, 51 years)
	Facilitators of diabetes care	Factors that enables diabetes care	The relationship between the patient and access to the healthcare team	"And in the message…I read it and I go show it to the doctor and ask her... and she explains it…" (Woman, 47 years)

	Barriers to diabetes care	Factors that negatively affect diabetes care	Situations of vulnerability such as material deprivation and difficulties in accessing healthy food or medical supplies or situations of psychosocial vulnerability linked to family disarticulation	“For me it’s fine, but the more support the better. You know why? Because I am a single parent, that is to say, mom and dad all in one…understand …?” (Woman, 47 years)
